# Correlations Between Novel Adiposity Indices and Electrocardiographic Evidence of Left Ventricular Hypertrophy in Individuals with Arterial Hypertension

**DOI:** 10.3390/jpm15060229

**Published:** 2025-06-02

**Authors:** Giulio Geraci, Pietro Ferrara, Francesco Pallotti, Rosario Le Moli, Vincenzo Calabrese, Valentina Paternò, Luca Zanoli, Antonina Giammanco, Alessandra Bellavia, Liliana Naro, Alessandra Sorce, Luigi La Via, Jacob George, Riccardo Polosa, Giuseppe Mulè, Caterina Carollo

**Affiliations:** 1 Department of Medicine and Surgery, “Kore” University of Enna, 94100 Enna, Italy; giulio.geraci@unikore.it (G.G.); rosario.lemoli@unikore.it (R.L.M.); vincenzo.calabrese@unikore.it (V.C.); polosa@unict.it (R.P.); 2 UOC Internal Medicine, Hospital Umberto I, ASP Enna, 94100 Enna, Italy; 3 Center for Public Health Research, University of Milan–Bicocca, 20900 Monza, Italy; 4 Laboratory of Public Health, IRCCS Istituto Auxologico Italiano, 20149 Milan, Italy; 5 Department of Clinical and Experimental Medicine, University of Catania, 95131 Catania, Italy; luca.zanoli@unict.it; 6 Unit of Internal Medicine, Department of Health Promotion, Mother and Child Care, Internal Medicine and Medical Specialties, University of Palermo, 90133 Palermo, Italy; antonina.giammanco@unipa.it (A.G.); alessandrabellavia93@gmail.com (A.B.); 7 UOC Internal Medicine, Hospital “S. Elias”, ASP Caltanissetta, 93100 Caltanissetta, Italy; liliana.naro@yahoo.it; 8 Unit of Nephrology, Department of Health Promotion, Mother and Child Care, Internal Medicine and Medical Specialties, University of Palermo, 90133 Palermo, Italy; alessandra.sorce@community.unipa.it (A.S.); giuseppe.mule@unipa.it (G.M.); caterina.carollo@unipa.it (C.C.); 9 Department of Anesthesia and Intensive Care 1, University Hospital Policlinico “G. Rodolico-San Marco”, 95131 Catania, Italy; luigilavia7@gmail.com; 10 School of Medicine, University of Dundee, Ninewells Hospital, Dundee DD1 9SY, UK; j.george@dundee.ac.uk; 11 Centre of Excellence for the Acceleration of HArm Reduction (CoEHAR), University of Catania, 95131 Catania, Italy; 12 European Society of Hypertension Excellence Center, Chair of Nephrology, University of Palermo, 90133 Palermo, Italy

**Keywords:** hypertension, obesity, cardiovascular risk, Sokolow-Lyon index, left ventricular hypertrophy, a body shape index, body roundness index, electrocardiography

## Abstract

**Background/Objectives:** Obesity is a key driver of cardiovascular disease (CVD), with central adiposity directly involved in adverse cardiac remodeling. Body mass index (BMI) is limited in capturing fat distribution and associated cardiovascular risk. Novel anthropometric indices, including A Body Shape Index (ABSI) and Body Roundness Index (BRI), may offer greater clinical value, but their relationship with electrocardiographic markers of left ventricular hypertrophy (LVH) remains underexplored. This study aims to assess the correlation between novel adiposity indices (ABSI and BRI) and electrocardiographic evidence of LVH, as measured by the Sokolow-Lyon Index (SLI), in individuals with arterial hypertension. **Methods**: 274 hypertensive patients were recruited, and BMI, ABSI, and BRI were calculated. LVH was assessed via SLI on 12-lead ECG. Participants were stratified by the SLI (≤35 mm vs. >35 mm) for statistical analyses. **Results**: Patients with a lower SLI showed significantly higher values of ABSI and BRI compared to those in higher SLI group, without differences in BMI. In the entire population, SLI was significantly and inversely correlated with both ABSI (r = −0.296, *p* < 0.001) and BRI (r = −0.238, *p* < 0.01), but not with BMI. Multivariate regression analysis confirmed ABSI (*p* = 0.013) and BRI (*p* = 0.038) as independent predictors of SLI, even after adjusting for age, blood pressure, renal function, and metabolic parameters. **Conclusions**: ABSI and BRI are inversely and independently associated with ECG-derived SLI in hypertensive individuals, suggesting that central adiposity may attenuate ECG voltages and obscure LVH detection. Incorporating novel adiposity indices into ECG interpretation may enhance diagnostic accuracy and risk stratification in obese and hypertensive populations. Longitudinal studies are needed to validate these findings and refine clinical algorithms.

## 1. Introduction

Obesity is a chronic, multifactorial disease that has reached epidemic proportions globally, posing a major challenge for public health systems. According to the World Health Organization (WHO), over 2.5 billion adults were overweight and more than 890 million obese in 2022. Obesity is strongly associated with increased cardiovascular morbidity and mortality, with central adiposity in particular contributing to adverse structural and functional cardiac remodeling [1,2].

The pathophysiology underlying obesity-related comorbidities is complex [3,4,5]. Excessive secretes bioactive molecules—such as adipokines, cytokines, and hormones—that disrupt metabolic balance and adversely affect cardiovascular and renal function. Central or visceral obesity, characterized by excessive fat deposition in the abdominal region, is associated with metabolic syndrome and type 2 diabetes, both of which increase cardiovascular risk [6]. The elevated release of free fatty acids from visceral adipose tissue promotes lipotoxicity, which impairs myocardial function and exacerbating heart failure. Hepatic steatosis, also common in obesity, contributes to dyslipidemia and systemic inflammation, further aggravating cardiovascular outcomes [7].

Hypertension, a major determinant of cardiovascular morbidity in obesity, arises from interrelated mechanisms. Excessive adiposity activates the sympathetic nervous system activation and disrupts the renin-angiotensin-aldosterone system (RAAS), causing vasoconstriction and sodium retention, which elevate blood pressure. Additionally, mechanical compression of the kidneys by adipose tissue results in parenchymal damage, impairing sodium excretion, further exacerbating hypertension. Together, obesity-induced hypertension and endothelial dysfunction accelerate atherosclerosis, increasing the risk of myocardial infarction, stroke, and peripheral artery disease [5,8,9].

A critical manifestation of obesity-related cardiovascular damage is left ventricular hypertrophy (LVH), as a consequence of increased cardiac workload, elevated circulating blood volume, and neurohormonal activation that lead to cardiac structural remodeling. LVH—which is also highly prevalent in hypertensive individuals—represents a key intermediate phenotype of target organ damage and is associated with poor cardiovascular outcomes. Over time, these changes predispose individuals to heart failure, which is a major contributor to heart failure hospitalizations [10,11].

Electrocardiography (ECG), despite its relatively low sensitivity and lower cardiovascular predictive value compared to echocardiography or cardiac magnetic resonance, remains a first-line tool for diagnosing LVH in routine clinical settings due to its accessibility and cost-effectiveness [12]. Over time, several ECG-based criteria and scores have been developed and validated for LVH detection. Among them, the Sokolow-Lyon criterion, in particular, is influenced by adiposity through a reduction of electrocardiographic voltages [13]. The low ECG sensitivity in detecting LVH persists even after ECG parameters are corrected for the body mass index (BMI), yet this correction does not substantially improve the diagnostic sensitivity of specific ECG criteria for LVH.

BMI, the most commonly used measure of obesity, is limited in its ability to capture fat distribution and is only modestly correlated with cardiovascular risk and LVH. Moreover, recent insights from the Lancet Diabetes & Endocrinology Commission highlight that traditional measures like body mass index (BMI) may not adequately capture individual health risks associated with obesity, underscoring the importance of comprehensive assessments beyond BMI to better understand and address the multifaceted health impacts of obesity [14]. As such, reliance on BMI may obscure the relationship between adiposity and subclinical cardiac damage. To address these limitations, novel anthropometric indices—such as A Body Shape Index (ABSI) and Body Roundness Index (BRI)—have been proposed. These indices incorporate waist circumference and body shape, potentially providing a more accurate representation of central obesity and its cardiovascular consequences [15,16,17,18]. Despite emerging evidence supporting the utility of ABSI and BRI in predicting cardiovascular outcomes, their relationship with electrocardiographic markers of LVH remains underexplored.

Therefore, the aim of our study is to analyze the relationships between novel anthropometric adiposity indices (ABSI and BRI) and left ventricular hypertrophy (LVH) assessed by ECG using the Sokolow-Lyon index (SLI) in individuals with arterial hypertension.

## 2. Materials and Methods

### 2.1. Study Population

This study included hypertensive individuals consecutively recruited from the European Centre of Excellence of the European Society of Hypertension at the University of Palermo, between 1 February 2024 and 30 September 2024. Hypertension was defined based on a prior clinical diagnosis and/or current use of antihypertensive medication, in line with the 2023 ESH Guidelines.

Patients meeting the following criteria were excluded from this research:

− class III obesity (BMI ≥ 40 kg/m^2^) or underweight (BMI < 20 kg/m^2^),
− secondary hypertension (renovascular, endocrine, or malignant),
− end-stage renal disease requiring renal replacement therapy,
− heart failure (NYHA class IV),
− history or presence of pleural/pericardial effusion,
− history of COPD, interstitial lung disease, thyroid disorders, or storage diseases,
− clinical signs of anasarca or cachexia,
− major non-cardiovascular comorbidities significantly affecting health status.

Exclusions were determined via detailed history, clinical documentation review, and outpatient evaluation. Secondary forms of hypertension were excluded through Duplex-Doppler renal ultrasound, serum electrolytes, plasma renin activity, and aldosterone concentration. The study was approved by the local ethics committee and conducted in accordance with the Declaration of Helsinki.

### 2.2. Study Design

A comprehensive medical history, a review of clinical records, and a physical examination were conducted for all participants. Current smokers were defined as individuals reporting regular cigarette use within the past year. Blood pressure was measured using a validated oscillometric device (Microlife WatchBP Office, Widnau, Switzerland) [19], and the final BP value was calculated as the mean of three consecutive readings obtained during a single office visit, following 5 min of rest, in accordance with the 2023 ESH guidelines [20]. All patients underwent anthropometric evaluation of adiposity indices and electrocardiographic evaluation. Routine laboratory tests included complete blood count, serum creatinine, fasting glucose, uric acid levels, and lipid profile. Analyses were performed using standardized methods on an automated analyser (Boehringer Mannheim for the Hitachi 911 system, Mannheim, Germany) as described elsewhere [21]. Serum uric acid was measured using the uricase/peroxidase method. Low-density lipoprotein cholesterol (LDL-C) was calculated using the Friedewald formula. The estimated glomerular filtration rate (eGFR) was derived using the Chronic Kidney Disease Epidemiology Collaboration (CKD-EPI) equation [22].

### 2.3. Anthropometric Indices of Adiposity

Body weight, height, and waist circumference (WC) were measured by a physician and used to calculate the body surface area (BSA), BMI, ABSI, and BRI using standardized formulas. BSA was calculated according to the DuBois formula, as BSA = 0.007184 × Weight 0.425 × Height 0.725 [23]. BMI was calculated as BMI = Weight/Height 2. ABSI, as proposed by Krakauer et al. [24], was computed as ABSI = WC/(BMI 2/3 × H 1/2), with WC in meters. BRI was derived from WC and height in meters, beginning with the calculation of eccentricity (ε), representing the degree of circularity of an ellipse (from 0 for a perfect circle to 1 for a vertical line), as previously described by Thomas et al. [25]. Values close to 1 are typical of individuals with a longitudinal body shape, whereas higher values indicate a more rounded body shape [25].

### 2.4. Sokolow Lyon Index (SLI)

Standard 12-lead ECG was performed in all subjects as part of normal clinical practice, according to international guidelines recommendations [20], and the Sokolow-Lyon index was calculated as ECG sign of LVH. Among the electrocardiographic criteria used for diagnosing LVH, the Sokolow-Lyon index was chosen since it is widely used due to its simplicity and reproducibility. Briefly, it is calculated by summing the amplitude of the S wave in lead V1 and the R wave in lead V5 or V6 (whichever is greater). A cut-off value > 35 mm is considered indicative of LVH. Although characterized by relatively low sensitivity, the index possesses high specificity, making it particularly valuable in epidemiological settings and population-based studies.

### 2.5. Statistical Analysis

Analysis was performed on the entire study population and subsequently stratified into two groups based on the SLI value (SLI ≤ or >35 mm). Continuous variables were reported as mean ± standard deviation (SD) or median and interquartile range (IQR), according to their distribution assessed using the Kolmogorov-Smirnov test. The latter were also log-transformed for parametric analyses. Categorical variables were presented as counts and/or percentages. Comparisons between groups were performed using Student’s *t*-test for continuous variables and Chi-square test (χ^2^) for categorical variables, applying Yates’ correction or Fisher’s exact when appropriate. For comparisons involving more than two groups, one-way ANOVA with Holm-Sidak post-hoc test was used for continuous variables, and χ^2^ test for categorical variables. Associations between variables were assessed using simple linear regression and Pearson correlation coefficients. These analyses were conducted in the overall cohort and within SLI subgroups (SLI ≤ or >35 mm). Differences in Pearson correlation coefficients between groups were tested using Fisher’s r-to-z transformation. Multivariate linear regression was conducted using SLI as the dependent variable. A backward stepwise approach was used, including covariates that were significant in univariate analysis. Independent variables included: age, sex, smoking status, eGFR, systolic (SBP) and diastolic blood pressure (DBP), ABSI (or, alternatively, BRI), BMI (or, alternatively, WC), fasting glucose, LDL-c, high-density lipoprotein cholesterol (HDL-c). Results were reported using both unstandardized (B) and standardized regression coefficients (β). A *p*-value < 0.05 was considered statistically significant. Analyses were performed using SPSS software, version 21.0 (IBM Corporation, New York, NY, USA). code.

## 3. Results

A total of 274 individuals with arterial hypertension were included in the final analysis. Participants were stratified into two groups based on the median SLI value: subgroup I with SLI ≤ 35 mm (*n* = 253) and subgroup II with SLI > 35 mm (n = 21). The main demographic, anthropometric, metabolic, and ECG characteristics of the overall study population and the two subgroups stratified by SLI are presented in Table 1. The two groups of patients did not differ significantly in the use of cardiovascular and antidiabetic drugs (all *p* > 0.05). Similarly, no differences in smoking habit, diabetes, or renal function (assessed as eGFR < 60 mL/min/1.73 m^2^) were observed between the two groups (all *p* > 0.05).

**Figure 1:**
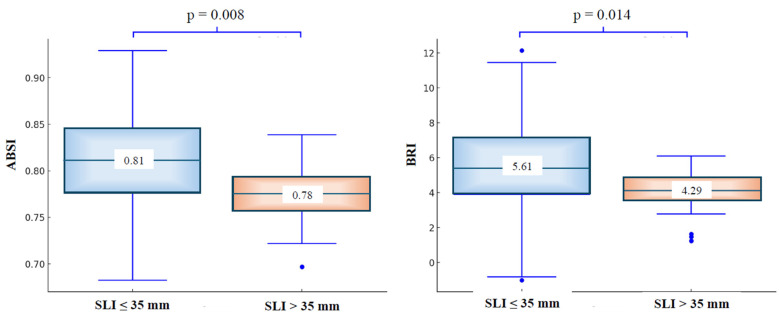
Mean values of ABSI and BRI in the two groups of patients divided by SLI (≤ or >35 mm).

As shown in Figure 1, patients with a lower SLI had significantly higher values of ABSI and BRI compared to those in the higher SLI group, although the two groups of patients did not significantly differ in BMI values.

The univariate correlations between SLI, anthropometric adiposity indices, and other clinical variables in the overall study population are presented in Table 2. SLI was significantly and inversely correlated with ABSI (r: −0.296; *p* < 0.001), and similar results were obtained with BRI (r: −0.238; *p* < 0.001). In contrast, no statistically significant correlation was observed between SLI and BMI. The difference in the strength of correlation between SLI and ABSI (or BRI) versus SLI and BMI was statistically significant (*p* = 0.05), as assessed using Fisher’s r-to-z transformation.

When we performed the multivariate linear regression analysis with a stepwise approach by considering SLI as the dependent variable, SLI was independently associated with ABSI, even after adjustment for all other covariates, including BMI or alternatively CV (*p* = 0.013). A similar independent association was observed when BRI replaced ABSI in the model (*p* = 0.038) (Table 3).

## 4. Discussion

This study explored the association between novel anthropometric indices of adiposity—ABSI and BRI—and electrocardiographic signs of LVH, as measured by the SLI, in a cohort of hypertensive patients. Our results reveal a significant and independent inverse association of both ABSI and BRI with SLI, even after adjusting for conventional confounders such as BMI, blood pressure, and renal function. A key observation is the strong correlation between these novel adiposity indices and traditional measures such as BMI and WC in hypertensive individuals. Strong linear correlations were noted between ABSI (or BRI) and BMI (or WC) across the entire study population.

These findings are consistent with previous studies: Krakauer et al. reported significant correlations between ABSI, BMI, and WC in the general population [24], while Thomas et al. found similar associations for BRI with conventional obesity indices [25]. Despite these relationships, BMI was not significantly correlated with SLI, highlighting its limitations in capturing the complexity of obesity-related cardiovascular risk. This supports existing evidence that BMI does not adequately reflect fat distribution or its cardiometabolic consequences, particularly in the context of central adiposity [14,26].

In contrast, ABSI and BRI—designed to reflect body shape and abdominal fat (Figure S1)—showed stronger associations with markers of subclinical cardiac remodeling, supporting their potential value in cardiovascular risk assessment [18,27,28,29]. The inverse correlation between ABSI and SLI may appear paradoxical, given the known association between adiposity and increased eft ventricular mass. However, this discrepancy may be explained by the physical and electrical interference of adipose tissue, which can attenuate ECG voltage transmission [30]. Excess truncal or visceral fat may reduce the surface voltages recorded by ECG, particularly in the leads used to calculate the SLI [31,32]. Moreover, the independent association between ABSI and SLI remained significant in multivariate analysis, even after adjusting for BMI, eGFR, blood pressure, and other relevant clinical variables. Substituting ABSI with BRI yielded comparable results, reinforcing their potential as modifiers of ECG interpretation in obese patients. Although our model explained only a moderate proportion of variance in SLI values (R^2^ = 0.217), this finding underscores the clinical relevance of these indices. Future research should include ROC analyses to further evaluate their diagnostic performance and assess whether they provide incremental value beyond traditional obesity metrics.

Although ECG criteria for LVH have been proposed with correction factors for BMI, these adjustments have generally yielded only modest improvements in diagnostic performance. Current ECG criteria for LVH, including BMI-adjusted thresholds, have shown only modest improvements in diagnostic accuracy. Our findings suggest that incorporating ABSI and BRI—indices that better reflect fat distribution—could enhance ECG-based detection of LVH in centrally obese hypertensive patients. By accounting for the voltage-attenuating effects of adiposity, these indices may help reduce false negatives and improve risk stratification when integrated into ECG interpretation algorithms [28,33].

Interestingly, we also observed that patients with lower SLI values had significantly higher ABSI, BRI, BMI, and WC—markers of central adiposity—compared to those with higher SLI values. This supports the hypothesis that increased adipose tissue may obscure the electrocardiographic detection of LVH, rather than indicating a truly lower prevalence of cardiac remodeling [31,34]. Our findings have important clinical implications. The SLI remains one of the most widely used ECG criteria for LVH detection due to its simplicity and cost-effectiveness [13]. However, its accuracy in individuals with elevated central adiposity may be compromised. In light of the global obesity epidemic and the rising prevalence of central obesity among hypertensive patients, our study supports the need to reconsider how adiposity influences ECG-based LVH diagnosis. Incorporating novel indices such as ABSI and BRI into ECG interpretation algorithms may improve risk stratification and help avoid false-negative results in this high-risk population.

Further supporting the physiological plausibility of these findings, a large rural Chinese study reported that BRI positively correlated with echocardiographic left ventricular mass index (LVMI) and predicted LVH [35]. This reinforces the idea that ABSI and BRI not only reflect true structural cardiac changes but may also interfere with ECG-based detection of those changes.

Several limitations should be acknowledged. First, although SLI is commonly used, it has limited sensitivity and may be suboptimal compared to imaging-based assessments of LVH, such as echocardiography or cardiac magnetic resonance. Notably, the diagnostic accuracy of echocardiographic criteria can also vary depending on comorbid conditions, particularly chronic kidney disease [36]. Second, LVH was assessed solely using SLI; other ECG-based criteria (e.g., Cornell voltage or Romhilt-Estes scores) or imaging modalities such as echocardiography were not systematically collected, which may limit the comprehensiveness of LVH detection. Future studies should explore and compare the diagnostic performance of alternative ECG-based indices, especially in obese and centrally obese populations. Third, our sample, although well-characterized, was relatively small and derived from a single center, potentially limiting generalizability. Fourth, body composition was assessed using anthropometric formulas rather than direct imaging or bioimpedance techniques, which might provide more accurate measures of visceral and subcutaneous fat. Finally, we did not analyze sex- and age-related differences in fat distribution and ECG voltages separately, which could have provided additional insights. Additionally, the lack of detailed data on individual pharmacological treatments limited our ability to evaluate their specific effects, as patients were only categorized as receiving therapy or not. This therapeutic heterogeneity may have contributed to variability in the results and should be addressed in future studies with more precise treatment information.

## 5. Conclusions

In conclusion, our study demonstrates that ABSI and BRI—novel adiposity indices that more accurately reflect body shape and visceral fat distribution—are independently and inversely associated with ECG-derived Sokolow–Lyon Index values in hypertensive individuals. These findings suggest that central adiposity may obscure the detection of LVH using standard ECG criteria, underscoring the need for adjusted interpretation strategies in obese patients. Future research, ideally longitudinal and multicentric, should investigate whether incorporating these indices into clinical algorithms can improve the diagnostic and prognostic accuracy of ECG-based LVH detection.

## Figures and Tables

**Table 1:** Demographic, anthropometric, and metabolic characteristics of the overall study population and of the groups divided by the median value of the Sokolow-Lyon Index.

	Total Cohort (n = 274)			SLI ≤ 35 mm (n = 253)			SLI > 35 mm (n = 21)			*p*-Value
Age (years)	52	±	15	54	±	15	45	±	14	<0.001
Male gender, n (%)	154 (56.2)			137 (54.2)			17 (80.9)			<0.001
Smoke, n (%)	94 (34.3)			89 (35.2)			5 (23.8)			0.685
*Clinical parameters*										
Diabetes, n (%)	72 (26.3)			68 (26.9)			4 (19)			0.485
eGFR < 60 mL/min/1.73 m^2^, n (%)	60 (21.9)			57 (22.5)			3 (14.3)			0.310
Sokolov-Lyon Index (mm)	22	±	7	21	±	5	38	±	4	<0.001
Clinic systolic BP (mmHg)	135	±	17	134	±	16	139	±	20	0.214
Clinic diastolic BP (mmHg)	81	±	12	81	±	12	88	±	14	0.007
Clinic mean BP (mmHg)	99	±	13	99	±	12	105	±	15	0.030
Clinic pulse pressure (mmHg)	53	±	13	53	±	13	51	±	12	0.453
Clinic heart rate (beats)	75	±	8	75	±	8	74	±	7	0.650
*Biochemical parameters*										
Fasting Glucose (mg/dL)	100.5	±	30.4	103.7	±	30.8	93.7	±	23.4	0.148
Uric Acid (mg/dL)	6.13	±	4.41	6.16	±	4.58	5.86	±	1.09	0.771
Total cholesterol (mg/dL)	189	±	41	189	±	41	196	±	48	0.432
LDL-c (mg/dL)	122	±	33	121	±	32	125	±	42	0.574
HDL-c (mg/dL)	49	±	12	49	±	12	57	±	17	0.003
Triglycerides (mg/dL)	111 (81–149)			111 (82–152)			107 (68–133)			0.771
Serum Creatinine (mg/dL)	1.04	±	0.61	1.03	±	0.59	1.19	±	0.73	0.253
eGFR (mL/min/1.73 m^2^)	83.7	±	26.7	82.8	±	25.7	94.9	±	35.4	0.046
*Anthropometric parameters*										
Weight (Kg)	79.3	±	17.3	79.5	±	17.8	77.3	±	10.6	0.575
Height (cm)	166	±	10	166	±	10	168	±	9	0.335
BSA (m^2^)	1.86	±	0.21	1.86	±	0.21	1.87	±	0.16	0.870
BMI (Kg/m^2^)	28.7	±	5.6	28.9	±	5.8	27.4	±	2.6	0.240
WC (cm)	99	±	15	99	±	16	91	±	8	0.016
BRI	5.51	±	2.37	5.61	±	2.42	4.29	±	1.19	0.014
ABSI	0.81	±	0.05	0.81	±	0.05	0.78	±	0.03	0.008
BMI ≥ 30 kg/m^2^, n (%)	78 (28.5)			83 (32.8)			5 (24.0)			0.436

SLI: Sokolov-Lyon Index eGFR: estimated Glomerular Filtration Rate; BP: Blood Pressure; LDL-c: Low Density Lipoprotein Cholesterol; HDL-c: High Density Lipoprotein Cholesterol; BSA: Body Surface Area; BMI: Body Mass Index; WC: waist circumference; BRI: Body Roundness Index; ABSI: A Body Shape Index.

**Table 2:** Univariate correlations between the Sokolow-Lyon Index, anthropometric adiposity indices, and other variables in the overall study population.

	SLI	ABSI	BRI	BMI	WC
	*r*	*r*	*r*	*r*	*r*
Age (years)	−0.308 ***	0.337 ***	0.139 *	0.003 ^NS^	0.076 ^NS^
Height (cm)	0.201 ***	−0.502 ***	−0.357 ***	−0.035 ^NS^	−0.043 ^NS^
Weight (Kg)	0.012 ^NS^	0.088 ^NS^	0.531 ***	0.837 ***	0.758 ***
Fasting glucose (mg/dL)	−0.144 *	0.160 *	0.152 *	0.151 *	0.160 *
Uric Acid (mg/dL)	0.142 *	0.005 ^NS^	0.025 ^NS^	0.160 *	0.183 **
Total Cholesterol (mg/dL)	−0.039 ^NS^	0.041 ^NS^	0.014 ^NS^	0.028 ^NS^	0.014 ^NS^
LDL-c (mg/dL)	−0.103 ^NS^	0.037 ^NS^	0.019 ^NS^	−0.003 ^NS^	0.002 ^NS^
HDL-c (mg/dL)	0.050 ^NS^	0.034 ^NS^	−0.062 ^NS^	−0.132 *	−0.130 *
HDL-c (mmol/L)	0.100 ^NS^	−0.006 ^NS^	−0.100 ^NS^	−0.140 *	−0.151 *
Triglycerides (mg/dL)	−0.036 ^NS^	−0.043 ^NS^	0.110 ^NS^	0.304 ***	0.239 ***
Serum Creatinine (mg/dL)	0.174 **	−0.026 ^NS^	0.030 ^NS^	0.004 ^NS^	0.016 ^NS^
eGFR (mL/min/1.73 m^2^)	−0.014 ^NS^	−0.151 *	−0.061 ^NS^	0.019 ^NS^	−0.008 ^NS^
Clinical Systolic BP (mmHg)	0.197 ***	−0.034 ^NS^	−0.065 ^NS^	−0.068 ^NS^	−0.053 ^NS^
Clinical Diastolic BP (mmHg)	0.260 ***	−0.160 *	−0.163 *	−0.098 ^NS^	−0.095 ^NS^
Clinical Mean BP (mmHg)	0.255 ***	−0.120 *	−0.134 *	−0.093 ^NS^	−0.085 ^NS^
Clinical Pulse Pressure (mmHg)	0.003 ^NS^	0.120 *	0.079 ^NS^	0.008 ^NS^	0.025 ^NS^
Clinical Heart Rate (beats/min)	0.134 *	−0.144 *	−0.110 ^NS^	−0.089 ^NS^	−0.105 ^NS^
SLI (mm)	1	−0.296 ***	−0.238 ***	−0.123 *	−0.182 **
BMI (kg/m^2^)	−0.121 ^NS^	0.458 ***	0.876 ***	1	0.934 ***
WC (cm)	−0.182 **	0.688 ***	0.917 ***	0.934 ***	1
ABSI	−0.296 ***	1	0.761 ***	0.458 ***	0.688 ***
BRI	−0.238 ***	0.761 ***	1	0.876 ***	0.917 ***

SLI: Sokolov-Lyon Index; ABSI: A Body Shape Index; BRI: Body Roundness Index; BMI: Body Mass Index; WC: Waist Circumference; LDL-c: Low Density Lipoprotein Cholesterol; HDL-c: High Density Lipoprotein Cholesterol; eGFR: estimated Glomerular Filtration Rate; BP: Blood Pressure. * *p* < 0.05; ** *p* ≤ 0.01; *** *p* ≤ 0.001; NS with *p* > 0.05.

**Table 3:** Multivariate linear regression analysis conducted in the overall population, considering Sokolow-Lyon Index (SLI) as the dependent variable and including ABSI along with other covariates in the model (see statistical section).

	Regression Coefficients				
	Non Standardized		Standardized		
	B	SE	β	t	*p*
Model (R^2^ = 0.217)					
Age	−0.187	0.033	−0.390	−5.61	<0.001
eGFR	−0.073	0.019	−0.254	−3.80	0.005
ABSI	−31.694	9.290	−0.197	−3.41	0.013
Systolic blood pressure	0.083	0.025	0.179	3.31	0.016

eGFR: estimated Glomerular Filtration Rate; SE: Standard Error; ABSI: A Body Shape Index.

## Data Availability

Data about this study will be made available on reasonable request.

## Supplementary Materials

The following supporting information can be downloaded at: https://www.mdpi.com/article/10.3390/jpm15060229/s1, Figure S1: Comparative Table of Anthropometric Indices (with Advantages).
