# Supplementary material for: Correlations Between Novel Adiposity Indices and Electrocardiographic Evidence of Left Ventricular Hypertrophy in Individuals with Arterial Hypertension
Source: J Pers Med. 2025 Jun 2;15(6):229. doi: 10.3390/jpm15060229 (PMC12194296; doi:10.3390/jpm15060229)
Supplement: Supplementary file 1 [file jpm-15-00229-s001.zip › jpm-3596992-supplementary.pdf]

Fig. S1 Comparative Table of Anthropometric Indices (with Advantages)

Index	Formula	Interpretation	Limitations	Advantages
**BMI (Body Mass Index)**	BMI = weight (kg) / height^2 (m^2)	Estimates general body fatness; widely used for obesity classification	Does not account for muscle mass, fat distribution, or body shape	Simple, quick, and widely accepted in clinical and epidemiological settings
**ABSI (A Body Shape Index)**	ABSI = WC / [BMI^(2/3) × height^(1/2)]	Measures abdominal adiposity adjusted for BMI and height	Complex formula; not widely adopted; may be less intuitive	Better predictor of mortality risk than BMI alone
**BRI (Body Roundness Index)**	BRI = 364.2 – 365.5 × √[1 – (WC / (2π))^2 / (0.5 × height)^2]	Estimates body shape and fat distribution; associated with cardiovascular risk	Requires accurate WC and height; not as commonly used clinically	Correlates well with body fat percentage and visceral adiposity
**WC (Waist Circumference)**	Measured directly (usually in cm) at the narrowest point between the ribs and hips	Indicates central obesity and visceral fat levels	Cutoffs vary by sex and ethnicity; doesn't consider overall body size or shape	Simple measure that directly assesses abdominal fat
